# Synthesis and Field Evaluation of the Sex Pheromone Analogues to Soybean Pod Borer *Leguminivora glycinivorella*

**DOI:** 10.3390/molecules171012140

**Published:** 2012-10-16

**Authors:** Dai-Hua Hu, Jun He, Yi-Wan Zhou, Jun-Tao Feng, Xing Zhang

**Affiliations:** 1 Biorational Pesticide Research & Development Center, Northwest A&F University, Yangling 712100, Shaanxi, China; Email: hejun6206@126.com (J.H.); Yiwan1021@163.com (Y.-W.Z.); jtfeng@126.com (J.-T.F.); 2 Shanxi Research Center of Biopesticide Engineering & Technology, Northwest A&F University, Yangling 712100, Shaanxi, China; 3 School of Chemical & Environment Science, Shaanxi University of Technology, Hanzhong 723000, Shaanxi, China

**Keywords:** *Leguminivora glycinivorella*, sex pheromone, synthesis, coupling reaction, attractive activity

## Abstract

In order to develop efficient lures for soybean pod borer *Leguminivora glycinivorella* (Matsumura) in China, (*E,E*)-8,10-dodecadienyl acetate (*EE-*8,10-12:Ac), the main component of the pheromone of *L. glycinivorella*, and 12 structurally-related compounds were synthesised in good overall yields, regiospecificities, and stereo-selectivities via coupling reactions catalysed by Li_2_CuCl_4_. The effect of different synthetic compounds, alone or in combination with *EE-*8,10-12:Ac, on numbers of captured *L. glycinivorella* males was evaluated. *EE-*8,10-12:Ac, (*E*)-10-dodecenyl acetate(*E-*10-12:Ac), (*E*)-8-dodecenol (*E-*8-12:OH), tetradecyl acetate (14:Ac), and (*Z*)-9-tetradecenyl acetate (*Z-*9-14:Ac) alone displayed different attractiveness to *L. glycinivorella* males. 14:Ac, *E-*8-12:OH, *E-*10-12:Ac, (*E,E*)-8,10-dodecadienal (*EE-*8,10-12:Ald), (*E*)-8-dodecenal (*E-*8-12:Ald), (*E*)-10-dodecenal (*E-*10-12:Ald) and *Z-*9-14:Ac all showed a synergistic effect to *EE-*8,10-12:Ac at certain dosages. The binary mixtures of *EE-*8,10-12:Ac and *E-*10-12:Ald, *Z-*9-14:Ac,14:Ac, *E-*8-12:Ald, *EE-*8,10-12:Ald, *E-*8-12:OH, or *E-*10-12:Ac in suitable ratios give 17.00-, 10.98-, 10.67-, 6.73-, 5.54-, 4.30- and 4.50-fold increases in trap catch, respectively, over the standard pheromone lure, and as novel pheromone blends, demonstrated potential use in pheromone traps to monitor or control *L. glycinivorella* populations in China.

## 1. Introduction

*Leguminivora glycinivorella* is one of the most destructive soybean pests in North China. Larvae feed mainly on the pod hulls, especially of young beans, severely reducing crop yield and quality. (*E,E*)-8,10-Dodecadienyl acetate (*EE-*8,10-12:Ac) is the main sex pheromone of *L. glycinivorella* [1]. In 2008 Wu reported the synthesis of *EE-*8,10-12:Ac by the use of Suzuki-Miyaura cross-coupling reaction between potassium (*E*)-1-propenyl trifluoroborate and (*E*)-9-iodo-1-(2-tetrahydropyranyloxy)-8-nonene in the presence of PdCl_2_(dppf), CH_2_Cl_2_ and Cs_2_CO_3_ [2]. However, this method suffers from high cost and difficulties associated with industrial scale-up. Alternatively, Wang *et al*. have implemented a field experiment on the attraction of *L. glycinivorella* using a mixture of *EE-*8,10-12:Ac and (*E*)-10-dodecenyl acetate (*E*10-12:Ac) at Gongzhuling and Dehui, Jilin Province, China [3]. A strong attraction to male moths by a mixture of *EE-*8,10-12:Ac and *E*10-12:Ac in a ratio of 2:5 was observed. Nevertheless, this reported pheromone lure does not work well in Heilongjiang Province, China. This discrepancy may attribute to the different habitats of *L. glycinivorella* in Heilongjiang and Jilin, the different climates (including temperature and humidity) and the planting structures.

Monounsaturated C12 and C14 alcohols, acetates and aldehydes are commonly found in lepidopteran pheromone glands [4], often as minor components, and some have been shown to synergize the attraction to the main pheromone component(s). For example, *EE-*8,10-12:Ald added at 0.002 mg to 2 mg *EE-*8,10-12:OH (the main component of the pheromone of the codling moth *Cydia pomonella*) increases the number of codling moth males caught in traps [5]. 

Our goal is to develop efficient lures for monitoring and controlling soybean pod borer population in China. Thus, *EE-*8,10-12:Ac and its chain-elongated, monounsaturated and aldehyde analogues were efficiently synthesised from the acetate and Grignard reagent via coupling reactions catalyzed by dilithium tetrachlorocuprate (Li_2_CuCl_4_), followed by evaluation of the effect of twelve different analogues, alone or in combination with *EE-*8,10-12:Ac, on numbers of *L. glycinivorella* males captured in traps. 

## 2. Results and Discussion

### 2.1. Chemistry

**Scheme 1:**
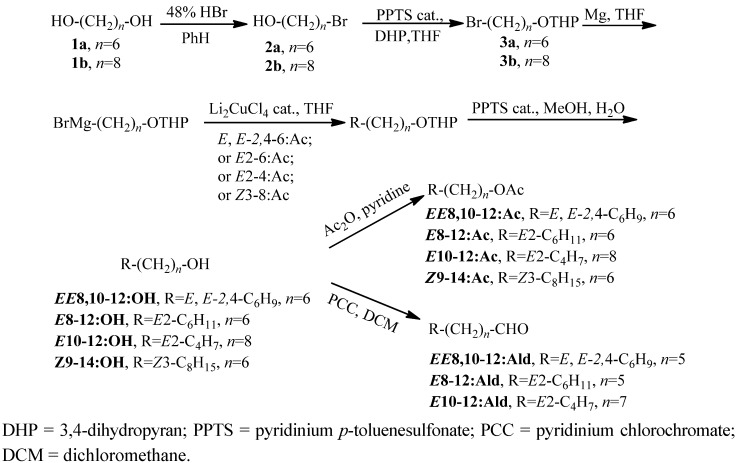
Synthetic route of *EE-*8,10-12:Ac and its analogues.

*EE-*8,10-12:OH, *EE-*8,10-12:Ac, (*E*)-8-dodecenol (*E-*8-12:OH), *E-*8-12:Ac, (*E*)-10-dodecenol (*E-*10-12:OH), (*E*)-10-dodecenyl acetate (*E-*10-12:Ac), *EE-*8,10-12:Ald, (*E*)-8-dodecenal (*E-*8-12:Ald), (*E*)-10-dodecenal (*E-*10-12:Ald), (*Z*)-9-tetradecenol (*Z-*9-14:OH), and (*Z*)-9-tetradecenyl acetate (*Z-*9-14:Ac) were synthesized with good overall yields, regiospecificities, and stereoselectivities as presented in Scheme 1 [6,7]. Dodecyl acetate (12:Ac) and tetradecyl acetate (14:Ac) were obtained by the acetylation of dodecenol and tetradecenol, respectively. Before field tests, all compounds were purified by column chromatography using silica gel without further isomerization as the configuration of the double bond can be maintained from the raw material of (*E,E*)-2,4-hexadienyl acetate (*EE-*2,4-6:Ac), *E*2-6:Ac, (*E*)-2-butenyl acetate (*E-*2-4:Ac), and (*Z*)-3-octenyl acetate (*Z-*3-8:Ac) to the corresponding target compounds.

### 2.2. Field Tests

In the present study, both, the choice of compound and dose significantly affected numbers of *L. glycinivorella* males captured in traps. Among the thirteen compounds and three doses combinations tested, traps baited with *EE-*8,10-12:Ac (0.1 mg), *E-*10-12:Ac (0.1 mg), *E-*10-12:Ac (0.5 mg), *E-*8-12:OH (0.01 mg), *E-*8-12:OH (0.1 mg), 14:Ac (0.1 mg), and *Z-*9-14:Ac (0.1 mg) had mean catches that differed significantly from controls (Table 1).

**Figure 1:**
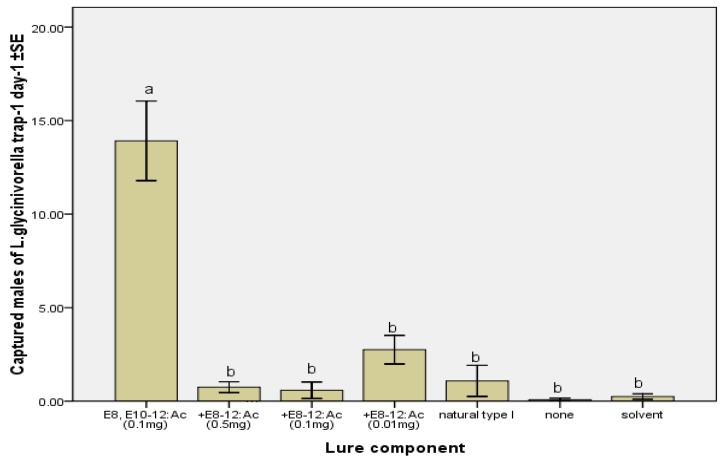
Mean (±SE) numbers of *L. glycinivorella* males captured per trap per day in traps baited with the binary blend of *EE-*8,10-12:Ac and *E-*8-12:Ac in different doses compared with control groups and natural type I [mixture of *EE-*8,10-12:Ac (30 ng) and *E*8-12:Ac (34 ng)] [8] in rubber septa. The field test was conducted in a soybean field in Harbin, China from 7 August to 17 August 2011. Bars with different letters are significantly different (ANOVA and Tukey’s HSD, *p* < 0.05).

The effect on catch when other synthetic compounds were combined with *EE*-8,10-12:Ac varied with the compound and dose. In 2006, it has been reported that *E-*8-12:Ac strongly inhibited the attractiveness of *EE-*8,10-12:Ac to *L. glycinivorella* in Japan [1]. When tested as a single compound, *E*-8-12:Ac was not attractive to *L. glycinivorella* males (Table 1). It significantly decreased the mean catch when added to traps baited with *EE*-8,10-12:Ac at dosage 0.05, 0.1, 0.01 mg/septum and as the mixture of natural type I (*EE*-8,10-12:Ac (30 ng) and *E-*8-12:Ac (34 ng)) [8] (Figure 1). Our results show that *E-*8-12:Ac is one of the inhibitors of the sex pheromone *EE-*8,10-12:Ac to *L. glycinivorella* in Heilongjiang Province, China. Thus, as pheromone antagonist, antipheromone and inhibitor, *E-*8-12:Ac showed broad prospects, especially when coupled with mating disruption of *L. glycinivorella*.

**Table 1:** Mean (±SE) numbers of *L. glycinivorella* males captured in traps baited with different synthetic compounds at three doses in a soybean field in Harbin, China, from 30 July to 6 August 2010.

Lure components	Dose (mg/lure)	No. of males captured/trap/day ^a^
	0.01	1.42 ± 0.22 defg
12:Ac	0.1	1.29 ± 0.18 defg
	0.5	1.58 ± 0.33 cdefg
	0.01	1.75 ± 0.21 cdefg
14:Ac	0.1	3.45 ± 0.47 c
	0.5	0.80 ± 0.41 efg
	0.01	0.33 ± 0.17 g
*EE-*8,10-12:OH	0.1	0.58 ± 0.08 g
	0.5	0.29 ± 0.04 g
	0.01	0.83 ± 0.18 efg
*EE-*8,10-12:Ac	0.1	10.56 ± 1.41 a
	0.5	1.17 ± 0.15 defg
	0.01	7.33 ± 0.86 b
*E-*8-12:OH	0.1	2.75 ± 0.21 cde
	0.5	0.74 ± 0.26 efg
	0.01	0.29 ± 0.08 g
*E-*8-12:Ac	0.1	0.29 ± 0.11 g
	0.5	0.33 ± 0.04 g
	0.01	0.67 ± 0.22 fg
*E-*10-12:OH	0.1	0.38 ± 0.12 g
	0.5	0.58 ± 0.18 g
	0.01	0.54 ± 0.04 g
*E-*10-12:Ac	0.1	10.25 ± 0.54 a
	0.5	6.58 ± 0.60 b
	0.01	1.19 ± 0.09 defg
*EE-*8,10-12:Ald	0.1	0.62 ± 0.19 fg
	0.5	1.14 ± 0.18 defg
	0.01	0.92 ± 0.15 defg
*E-*8-12:Ald	0.1	0.50 ± 0.07 g
	0.5	1.58 ± 0.18 cdefg
	0.01	0.84 ± 0.10 efg
*E-*10-12:Ald	0.1	1.04 ±0.11 defg
	0.5	0.88 ± 0.26 defg
	0.01	0.62 ± 0.21 fg
*Z-*9-14:OH	0.1	0.88 ± 0.14 defg
	0.5	0.87 ± 0.33 defg
	0.01	1.08 ± 0.36 defg
*Z-*9-14:Ac	0.1	2.62 ± 0.14 cdef
	0.5	2.87 ± 0.44 cd
Control (blank)	0	0.08 ± 0.04 g
Control (solvent)	0	0.15 ± 0.07 g

^a^ Catches followed by different letters within one column are significantly different (ANOVA and Tukey’s HSD, *p* < 0.05).

**Figure 2:**
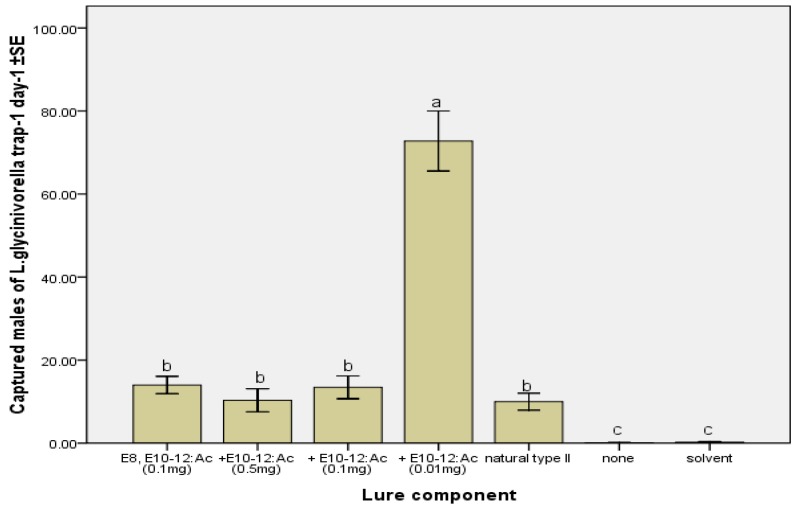
Mean (±SE) numbers of *L. glycinivorella* males captured per trap per day in traps baited with the binary blend of *EE-*8,10-12:Ac and *E-*10-12:Ac in different doses compared with control groups and natural type II (mixture of *EE-*8,10-12:Ac and *E-*10-12:Ac in a ratio of 2:5) [3] in rubber septa. The field test was conducted in a soybean field in Harbin, China from 7 August to 17 August 2011. Bars with different letters are significantly different (ANOVA and Tukey’s HSD, *p* < 0.05).

The combination of *EE-*8,10-12:Ac and *E-*10-12:Ac in a suitable ratio enhanced the attractiveness to *L. glycinivorella* males in field applications compared with *EE-*8,10-12:Ac administered alone. Wang showed that lures baited with a mixture of *EE-*8,10-12:Ac and *E-*10-12:Ac in a ratio of 2:5 (natural type II) were extremely attractive to *L. glycinivorella* at Gongzhuling and Dehui, Jilin Province, China [3]. Contrarily, the present work, which was carried out in Harbin, Heilongjiang Province, China, observed a decreased attractiveness with a number of captured male moths of only 4.0 trap^−1^ day^−1^ when using the mixture of natural type II compared with singly administered *EE-*8,10-12:Ac (10.21 trap^−1^ day^−1^). However, a significantly enhanced attractiveness (55 trap^−1^ day^−1^) of male moth was observed for a molecular ratio of 10:1 for a binary blend of *EE-*8,10-12:Ac and *E-*10-12:Ac (Figure 2). This result suggests the possibility that the sex pheromone of this species varies geographically. Nevertheless, a 10:1 synthetic mixture of *EE-*8,10-12:Ac and *E-*10-12:Ac could be used to efficiently monitor and/or control the pod borer field population in China.

**Figure 3:**
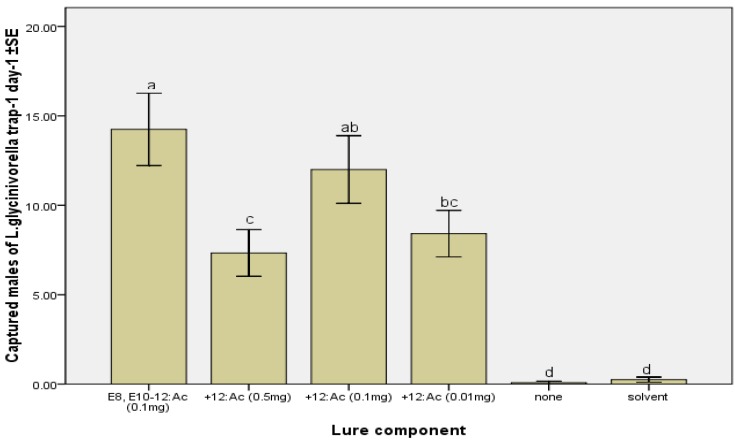
Mean (±SE) numbers of *L. glycinivorella* males captured per trap per day in traps baited with the binary blend of *EE-*8,10-12:Ac and 12:Ac in different doses compared with control groups in rubber septa. The field test was conducted in a soybean field in Harbin, China from 7 August to 17 August 2011. Bars with different letters are significantly different (ANOVA and Tukey’s HSD, *p* < 0.05).

Compound 12:Ac has been detected in a gland extract of *L. glycinivorella* pheromone, and the GC-EAD showed that it was antennally active [1]. When tested as a single compound, 12:Ac alone was not attractive to *L. glycinivorella* males (Table 1), and significantly decreased mean catch when added to traps baited with *EE-*8,10-12:Ac at doses of 0.01 and 0.5 mg per septum (Figure 3). Interestingly, however, doses of 0.5 and 0.01 mg of 12:Ac were inhibitory, but an intermediate dose of 0.1 mg was not. This can be explained as follows: generally, only the concentration of pheromone in the field which is similar to the concentration of pheromone in natural female moth can be received as a signal and effectively attract the male moth. Additionally, field catches can be affected by various factors, such as humidity, temperature, and the wind force, *etc*.

**Figure 4:**
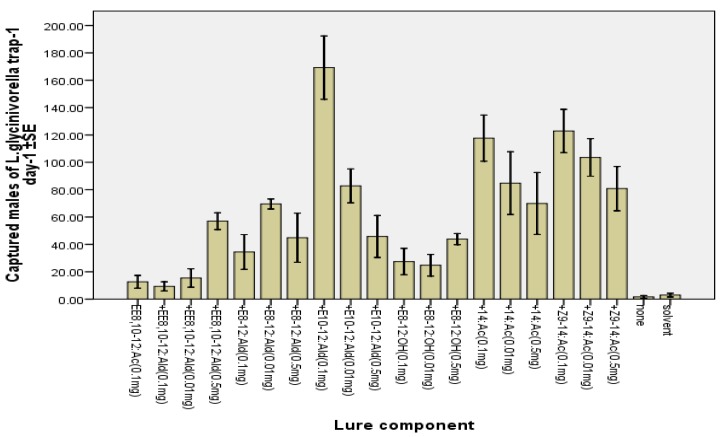
Attraction of *L. glycinivorella* males in traps baited with the binary blend of *EE-*8,10-12:Ac (0.1 mg) and nine different synthetic compounds at three doses (0.01, 0.1, and 0.5 mg) in a soybean field in Harbin, China, from 11 August to 17 August 2011. Catches followed by different letters within one column are significantly different at *p* = 5% by Tukey’s HSD [capture data were transformed to log(x + 1) before performing analysis].

Up to now, there were no reports on *E-*8-12:OH being attractive to *L. glycinivorella*. In the present study, *E-*8-12:OH alone displayed a significant attractiveness to the borer males (Table 1), and also showed a synergistic effect with *EE-*8,10-12:Ac (Figure 4). 

It has been reported that the compounds 14:Ac and *Z*9-14:Ac are pheromone components of two other soybean pod pests, *Etiella behrii* [9] and *Etiella zinckenella* [10,11,12]. In the present study, 14:Ac and *Z-*9-14:Ac were attractive to *L. glycinivorella* males when administrated individually compared to the controls (Table 1). However, the addition of a small amount of 14:Ac and *Z-*9-14:Ac to *EE-*8,10-12:Ac significantly enhanced the attractiveness of *EE-*8,10-12:Ac to *L. glycinivorella* males (Figure 4). 

Up to now, there has been no report on *EE-*8,10-12:Ald, *E-*8-12:Ald and *E-*10-12:Ald being attractants of *L. glycinivorella*. In the present study, weak attractiveness of these three compounds has been observed when administrated individually (Table 1). Surprisingly, the addition of a small amount of these three compounds significantly enhanced the activity of *EE-*8,10-12:Ac (Figure 4), especially the compound *E-*10-12:Ald at 0.1 mg dosage. These significant synergistic effects of *EE-*8,10-12:Ald, *E-*8-12:Ald and *E-*10-12:Ald to *EE-*8,10-12:Ac indicated their potential usefulness in the biological control of *L. glycinivorella* males in China. This work also shed light on the application of this kind of compounds as sex attractants of other Lepidopteran insects. No other moth species were captured in significant numbers during the field tests.

## 3. Experimental

### 3.1. General

NMR spectra were recorded at 500 MHz and 125 MHz for ^1^H and ^13^C, respectively, on a Brüker, ADVANCE III instrument (Brüker BioSpin AG, Fällanden, Switzerland) in CDCl_3_ solution with Me_4_Si as internal standard. The GC/MS analysis of the synthetic compounds was performed on a Thermo Trace gas chromatograph (Thermo Finnigan, Miami, FL, USA) coupled to Polaris Q mass spectrometer (Thermo Finnigan, Miami, FL, USA) under the following analytical conditions: HP-5 capillary column (15 m × 0. 53 mm × 0. 25 μm film thickness); helium (0.5 mL/min); programmed temperature 50–220 °C (7 °C/min); injector temperature (250 °C) and interface (230 °C); ionization energy, 70 eV; scan range, 30–500 amu; scan time, 1 s. All solvents were purified by standard procedures. GF_254_ TLC plates are used for the monitoring of reaction, and silica gel of 200–300 mesh is used for the column chromatography (Qingdao Haiyang Chemical Co., Qingdao, China). The acetate and aldehyde derivatives were obtained by the acetylation and pyridinium chlorochromate (PCC) oxidation of the corresponding alcohols, respectively [13,14].

*(E,E)-8,10-Dodecadienyl acetate (*EE*-8,10-12:Ac).* Yield 88%; Purity: 98%; MS (*m/z*, M^+^): 224; ^1^H-NMR (CDCl_3_), δ: 1.3–1.38 (m, 8H), 1.63 (t, *J* = 7.0 Hz, 2H), 1.73 (t, *J* = 7.0 Hz,3H), 2.04 (q, *J* = 7.0 Hz, 5H), 3.63 (t, *J* = 7.0 Hz, 2H), 5.51–5.59 (m, 2H), 5.96–6.02 (m, 2H); ^13^C-NMR (CDCl_3_), δ: 171.2, 132.0, 131.7, 130.2, 126.7, 64.6, 32.5, 29.3, 29.1, 29.0, 28.5, 25.8, 21.0, 18.0.

*(E)-8-Dodecenol (*E*-8-12:OH).* Yield 71%; Purity: 98%; MS (*m/z*, M^+^): 184; ^1^H-NMR (CDCl_3_), δ: 0.84–0.90 (m, 3H), 1.26–1.33 (m, 10H), 1.56 (dd, *J*_1_ = 3.5Hz, *J*_2_ = 7.0 Hz, 2H), 1.95 (d, *J* = 6.5 Hz, 4H), 3.63 (t, *J* = 6.5 Hz, 2H), 4.12 (br, 1H), 5.38 (t, *J* = 3.5 Hz, 2H); ^13^C-NMR (CDCl_3_), δ: 130.4, 130.2, 63.0, 34.7, 32.7, 32.5, 29.5, 29.2, 29.0, 25.7, 22.7, 13.6.

*(E)-8-Dodecenyl acetate (*E*-8-12:Ac).* Yield 88%; Purity: 97%; MS (*m/z*, M^+^): 226; ^1^H-NMR (CDCl_3_), δ: 0.84–0.89 (m, 3H), 1.19–1.42 (m, 12 H), 1.61 (dd, *J*_1_ = 3.5 Hz, *J*_2_ = 7.0 Hz, 2H), 1.93-1.96 (m, 2H), 2.04 (s, 3H), 4.05 (d, *J* = 7.0 Hz, 2H), 5.38 (t, *J* = 3.5 Hz, 2H); ^13^C-NMR (CDCl_3_), δ: 171.2, 130.4, 130.2, 64.6, 34.7, 32.5, 29.5, 29.1, 28.9, 28.6, 25.8, 22.7, 21.0, 13.6.

*(E)-10-Dodecenyl acetate (*E*-10-12:Ac).* Yield 87%; Purity: 98%; MS (*m/z*, M^+^): 226; ^1^H-NMR (CDCl_3_), δ: 0.85–0.89 (m, 3H), 1.20–1.31 (m, 12H), 1.62 (dd, *J*_1_ = 3.5Hz, *J*_2_ = 7.0 Hz, 2H), 1.95–1.96 (m, 2H), 2.04 (s, 3H), 4.05 (t, *J* = 7.0 Hz, 2H), 5.41 (t, *J* = 3.5 Hz, 2H); ^13^C-NMR (CDCl_3_), δ: 171.2, 131.6, 124.5, 64.6, 32.5, 29.6, 29.5, 29.4, 29.2, 29.1, 28.6, 26.9, 21.0, 17.9.

*(E,E)-8,10-Dodecadienal (*EE*-8,10-12:Ald).* Yield 85%; Purity: 97%; MS (*m/z*, M^+^): 180; ^1^H-NMR (CDCl_3_), δ: 1.34–1.41 (m, 6H), 1.63–1.70 (m, 2H), 1.75 (d, *J* = 7.0 Hz, 3H), 2.05–2.09 (m, 2H), 2.44 (t, *J* = 7.0 Hz, 2H), 5.54–5.62 (m, 2H), 5.98~6.07 (m, 2H), 9.78 (s, 1H); ^13^C-NMR (CDCl_3_), δ: 202.9, 131.8, 131.6, 130.4, 126.8, 43.8, 32.4, 29.1, 29.0, 28.8, 22.0, 18.0.

*(E)-8-Dodecenal (*E*-8-12:Ald).* Yield 85%; Purity: 97%; MS (*m/z*, M^+^): 182; ^1^H-NMR (CDCl_3_), δ: 0.91 (t, *J* = 7.0 Hz, 3H), 1.24–1.43 (m, 6H), 1.63–1.67 (m, 4H), 1.96–2.00 (m, 4H), 2.44 (t, *J* = 7.0 Hz, 2H), 5.41–5.42 (m, 2H), 9.79 (s, 1H); ^13^C-NMR (CDCl_3_), δ: 202.9, 130.3, 130.2, 43.9, 34.7, 32.4, 29.3, 29.0, 28.8, 22.7, 22.0, 13.6.

*(E)-10-Dodecenal (*E*-10-12:Ald).* Yield 84%; Purity: 97%; MS (*m/z*, M^+^): 182; ^1^H-NMR (CDCl_3_), δ: 1.27–1.30 (m, 12H), 1.61 (d, *J* = 7.0 Hz, 3H), 1.96 (s, 2H), 2.42 (t, *J* = 7.0 Hz, 2H), 5.36–5.41 (m, 2H), 9.76 (s, 1H); ^13^C-NMR (CDCl_3_), δ: 203.0, 131.6, 124.6, 43.9, 32.5, 29.5, 29.3, 29.2, 29.1, 29.0, 22.0, 17.9.

*(Z)-9-Tetradecenyl acetate (*Z*-9-14:Ac).* Yield 87%; Purity: 97%; MS (*m/z*, M^+^): 254; ^1^H-NMR (CDCl_3_), δ: 0.90 (t,*J* = 7.0 Hz, 3H), 1.32–2.04 (m, 21H), 2.38 (q, *J* = 7.0 Hz, 2H), 4.06 (t, *J* = 7.0 Hz, 2H), 5.35 (d, *J* = 7.5 Hz, 1H), 5.48 (d, *J* = 7.5 Hz, 1H); ^13^C-NMR (CDCl_3_), δ: 171.1, 132.9, 124.2, 64.0, 32.7, 31.8, 31.6, 30.7, 27.0, 26.8, 25.4, 22.6, 22.3, 21.0, 14.0, 13.9.

### 3.2. Field Trapping Experiments

Trapping experiments were carried out during July and August 2010 and 2011 in two soybean fields separated 30 km in Harbin, Heilongjiang Province, China during the flight period of *L. glycinivorella* males. Mean temperature, relative humidity, and wind speed during the trapping period were 25 °C, 50%, and 6.0 ms^−^^1^ in 2010, and 23 °C, 60%, and 6.5 ms^−^^1^ in 2011. Synthetic chemicals were dissolved in ca. 100 μL of dichloromethane and impregnated into rubber septa (0.3 g, 10 mm outside diameter × 16 mm height; Xi’an Dizhai Co., Shaanxi, China) at various doses (see below). The solvent was evaporated at room temperature overnight. A water pot (green, 24 cm diameter × 10 cm depth, Weilu plastic Co., Qingdao, Shandong, China) 3/4 filled with water and 0.5% detergent was employed as the trap. Traps in one replicate were hung from a wire, suspended 1–2 cm above the surface of the water in the trap which was hung on a tripod 1 m above the soil surface at ca. 13 m from each other in a line. Six replicates were used for each treatment. The treatments were completely randomized design and the positions of the traps were rotated to eliminate the position effect. Insects captured on the traps were removed every morning and identified, sexed, and catalogued. The water in the pot was replenished every week. Two types of controls were included: rubber septa impregnated with 100 µL dichloromethane as a solvent control, and untreated rubber septa. In experiment 1, the effect of dose (0.01, 0.1, and 0.5 mg per lure) on capture of *L. glycinivorella* males in traps baited with thirteen different synthetic compounds was tested from 30 July to 6 August 2010. In experiment 2, we tested nine different synthetic compounds (12:Ac, 14:Ac, *E-*8-12:OH, *E-*8-12:Ac, *E-*10-12:Ac, *EE-*8,10-12:Ald, *E-*8-12:Ald, *E-*10-12:Ald and *Z-*9-14:Ac) at three doses (0.01, 0.1, and 0.5 mg) added to traps baited with *EE-*8,10-12:Ac (0.1 mg) for their effect on numbers of *L. glycinivorella* males captured in traps. This experiment was conducted in the period from 7 August to 17 August 2011. 

### 3.3. Statistical Analysis

Data were analysed using ANOVA. Tukey’s HSD were used to determine significant differences between treatments. Capture data were transformed by log(x + 1) before performing analysis and statistically evaluated by ANOVA followed by Tukey’s HSD in lures baited with *EE-*8,10-12:Ac, and its binary mixture with *EE-*8,10-12:Ald, *E-*8-12:Ald, *E-*10-12:Ald, *Z-*9-14:Ac, *E-*8-12:OH, or 14:Ac. All statistical procedures were conducted using SPSS 16.0 for Windows. In all cases, the accepted level of significance was 5%.

## 4. Conclusions

In summary, *EE-*8,10-12:Ac, the main component of the pheromone of *L. glycinivorella*, and 12 of its structurally-related analogues were conveniently synthesised in good overall yields, regiospecificities, and stereoselectivities. Among the synthetic compounds, *E-*10-12:Ald, or binary mixtures of *Z-*9-14:Ac, 14:Ac, *E-*8-12:Ald, *EE-*8,10-12:Ald, *E-*8-12:OH, *E-*10-12:Ac with *EE-*8,10-12:Ac in appropriate ratios gave 17.00-, 10.98-, 10.67-, 6.73-, 5.54-, 4.30- and 4.50-fold increases in trap catch, respectively, over the lure baited with *EE-*8,10-12:Ac alone. These novel pheromone blends could be used in pheromone traps as biological control of *L. glycinivorella* populations in China.
